# Supplementary material for: Supplier-origin mouse microbiomes significantly influence locomotor and anxiety-related behavior, body morphology, and metabolism
Source: Commun Biol. 2021 Jun 10;4:716. doi: 10.1038/s42003-021-02249-0 (PMC8192786; doi:10.1038/s42003-021-02249-0)
Supplement: Supplementary file 2 — Supplementary Information [file 42003_2021_2249_MOESM2_ESM.pdf]

Supplementary Information

Supplier-origin mouse microbiomes significantly influence locomotor and anxiety-related behavior, body morphology, and metabolism

Short title: Supplier-origin mouse microbiomes, behavior, and body morphology

Aaron C. Ericsson[1,2]*, Marcia L. Hart[3], Jessica Kwan[4], Louise Lanoue[5], Lynette R Bower[5], Renee Araiza[5,6], K. C. Kent Lloyd[5,6,7,8], Craig L. Franklin[1,2]*

[1]University of Missouri Metagenomics Center (MUMC), Department of Veterinary Pathobiology, College of Veterinary Medicine, University of Missouri (MU), Columbia, MO, USA

[2]MU Mutant Mouse Resource and Research Center (MU MMRRC), Columbia, MO, USA

[3]IDEXX BioAnalytics, Columbia, MO, USA

[4]School of Veterinary Medicine, University of California (UC), Davis, CA, USA

[5]Mouse Metabolic Phenotyping Center (MMPC) at UC Davis, Davis, CA, USA

[6]Mutant Mouse Resource and Research Center at UC Davis, Davis, CA, USA

[7]UC Davis Mouse Biology Program (MBP), Davis, CA, USA

[8]Department of Surgery, School of Medicine, UC Davis, Sacramento, CA, USA

***Corresponding authors:** ericssona@missouri.edu (ACE), franklinc@missouri.edu (CLF)

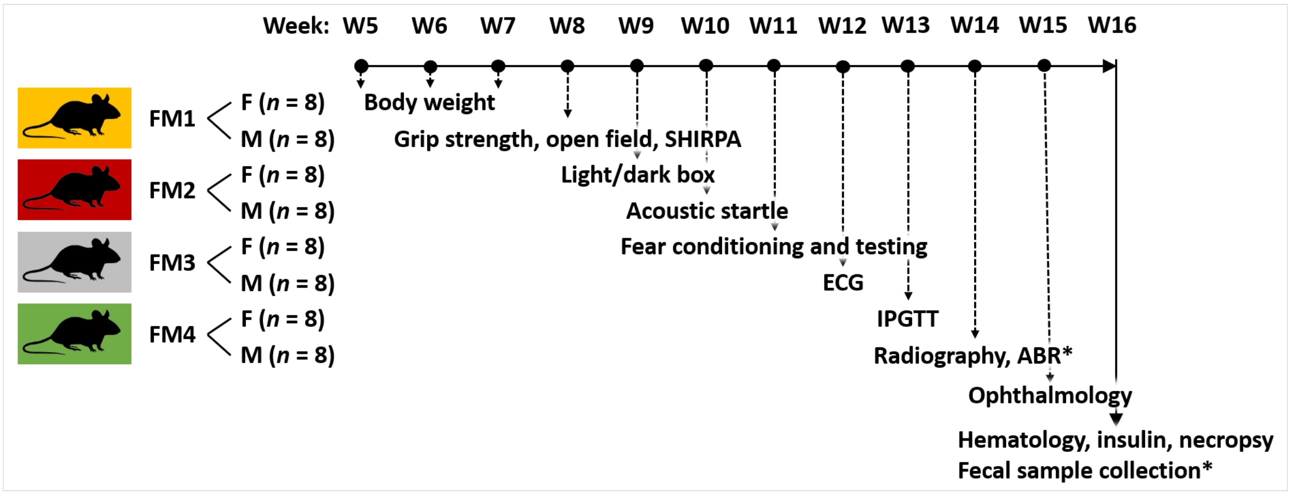

S1 Fig. Experimental design and phenotyping timeline

Schematic diagram of the experimental groups and timeline for the phenotyping assays and necropsy. Asterisk denotes that phenotyping was performed on a subset of mice. Fecal samples collected from experimental mice (n = 8/sex/FM) were compared retrospectively to data from colonies used to generate these mice (n = 12/sex/FM).

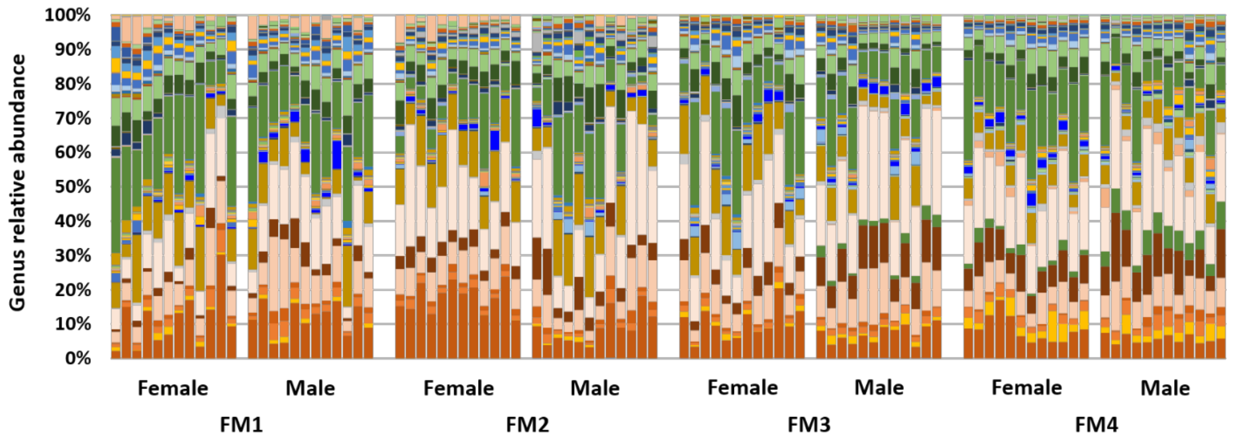

S2 Fig. Genus-level diversity in parent colonies used to generate mice used in phenotyping tests

Stacked bar chart showing taxonomic diversity, at the level of genus, in female and male CD-1 mice (*n* = 12/sex/FM) harboring one of four supplier-origin fecal microbiomes (FM1 through FM4), and representing the parent colonies prior to shipping.

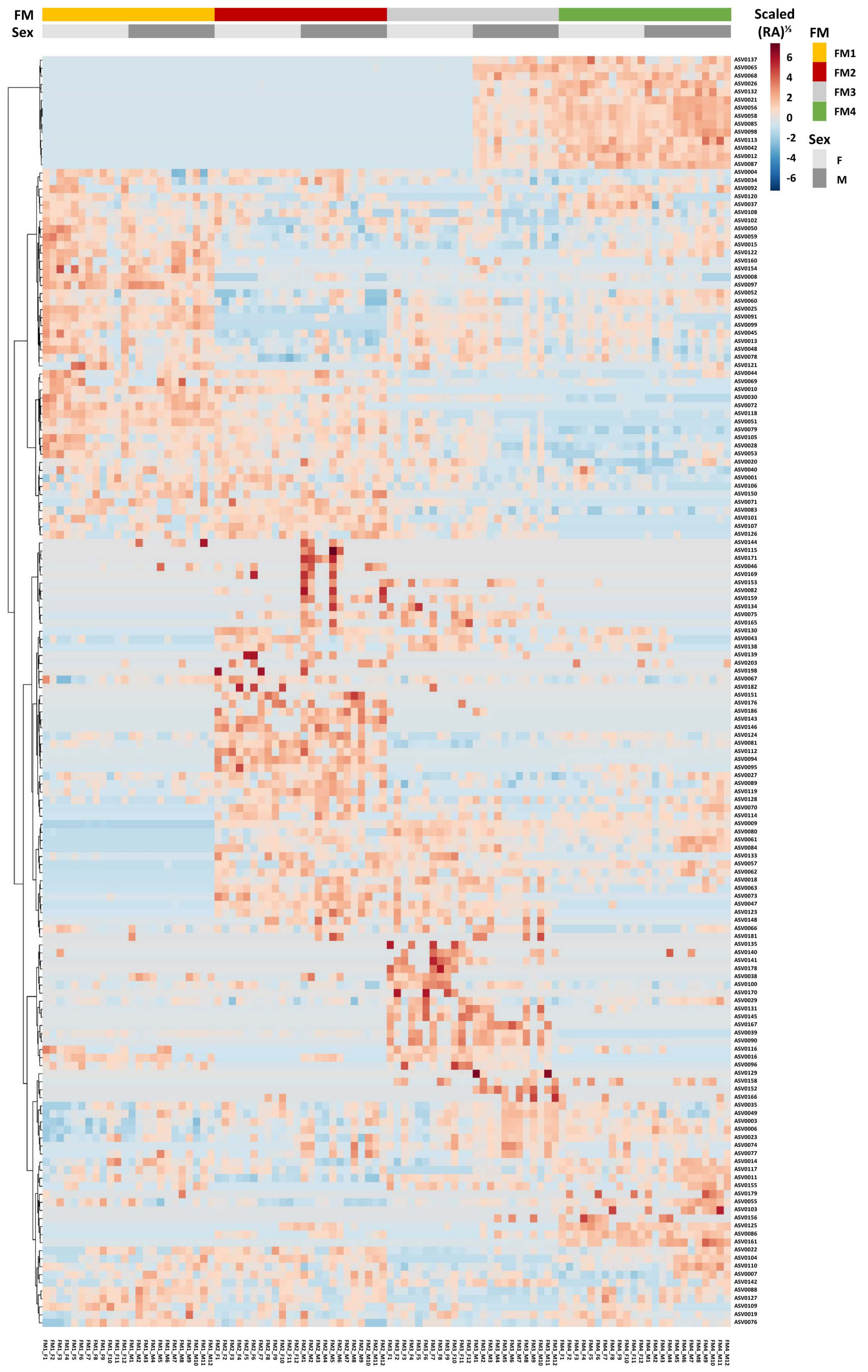
FM
Sex
Scaled
(RA)⅓
6
4
2
0
-2
-4
-6
FM
FM1
FM2
FM3
FM4
Sex
F
M
ASV0137
ASV0065
ASV0068
ASV0026
ASV0132
ASV0021
ASV0056
ASV0058
ASV0085
ASV0098
ASV0113
ASV0042
ASV0012
ASV0087
ASV0004
ASV0034
ASV0092
ASV0120
ASV0037
ASV0108
ASV0102
ASV0050
ASV0059
ASV0015
ASV0122
ASV0160
ASV0154
ASV0008
ASV0097
ASV0052
ASV0060
ASV0025
ASV0091
ASV0099
ASV0045
ASV0013
ASV0048
ASV0078
ASV0121
ASV0044
ASV0069
ASV0010
ASV0030
ASV0072
ASV0118
ASV0051
ASV0079
ASV0105
ASV0028
ASV0053
ASV0020
ASV0040
ASV0001
ASV0106
ASV0150
ASV0071
ASV0083
ASV0101
ASV0107
ASV0126
ASV0144
ASV0115
ASV0171
ASV0046
ASV0169
ASV0153
ASV0082
ASV0159
ASV0134
ASV0075
ASV0165
ASV0130
ASV0043
ASV0138
ASV0139
ASV0203
ASV0198
ASV0067
ASV0182
ASV0151
ASV0176
ASV0186
ASV0143
ASV0146
ASV0124
ASV0081
ASV0112
ASV0094
ASV0095
ASV0027
ASV0089
ASV0119
ASV0128
ASV0070
ASV0114
ASV0009
ASV0080
ASV0061
ASV0084
ASV0133
ASV0057
ASV0062
ASV0018
ASV0063
ASV0073
ASV0047
ASV0123
ASV0148
ASV0066
ASV0181
ASV0135
ASV0140
ASV0141
ASV0178
ASV0038
ASV0100
ASV0170
ASV0029
ASV0131
ASV0145
ASV0167
ASV0039
ASV0090
ASV0116
ASV0016
ASV0096
ASV0129
ASV0158
ASV0152
ASV0166
ASV0035
ASV0049
ASV0003
ASV0006
ASV0023
ASV0074
ASV0077
ASV0014
ASV0117
ASV0011
ASV0155
ASV0179
ASV0055
ASV0103
ASV0156
ASV0125
ASV0086
ASV0161
ASV0022
ASV0104
ASV0110
ASV0007
ASV0142
ASV0088
ASV0127
ASV0109
ASV0019
ASV0076

S3 Fig. FM- and sex-associated features within the microbiota

Heatmap showing all amplicon sequence variants (ASVs) with significant FM- or sex-dependent differences in relative abundance (RA), clustered according to taxonomic RA, see also **Supplementary Data 1** for taxonomic identity of ASVs and *p* and F values from two-way ANOVA of cube-root-transformed data.

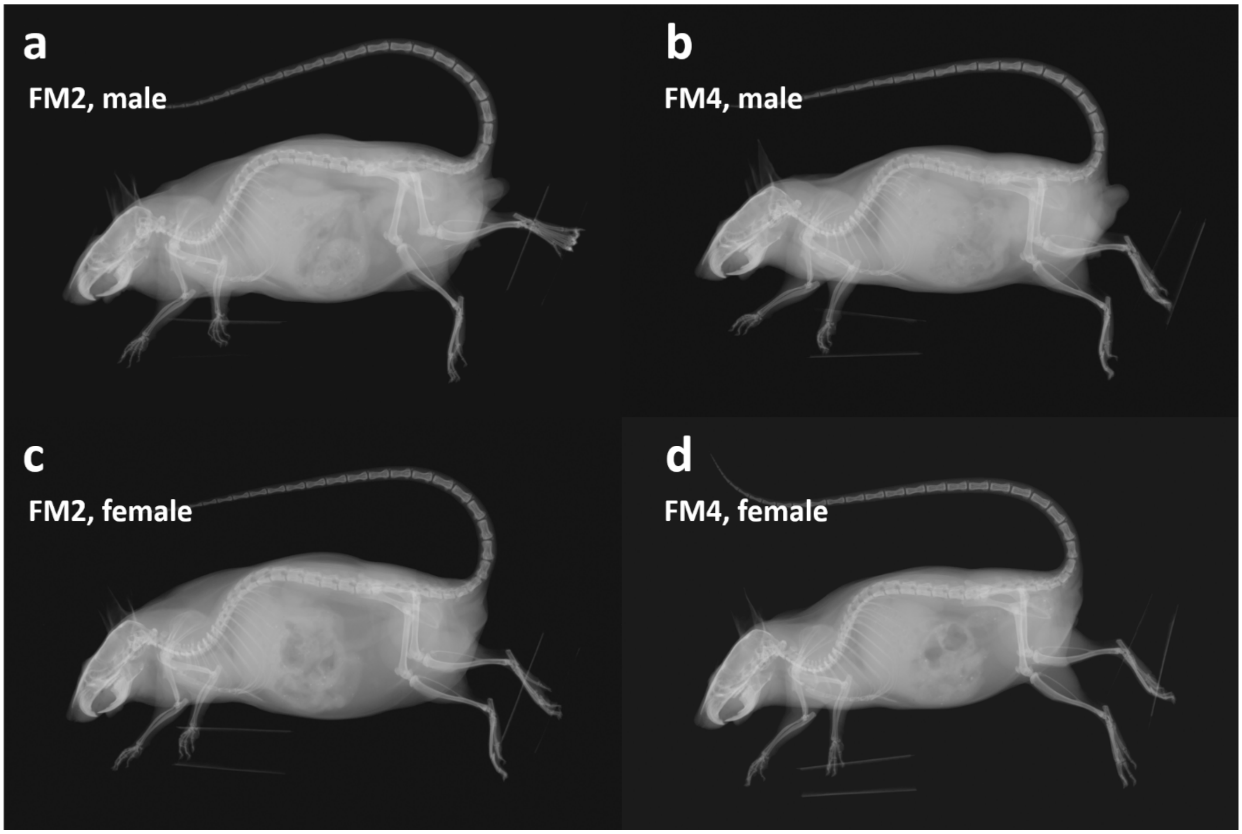

S4 Fig. Lateral radiographic views of the heaviest male and female mice within FM2 or FM4

Lateral radiographic views of (a) the heaviest male mouse colonized with FM2, (c) the heaviest male mouse colonized with FM4, (c) the heaviest female mouse colonized with FM2, and (d) the heaviest female mouse colonized with FM4.

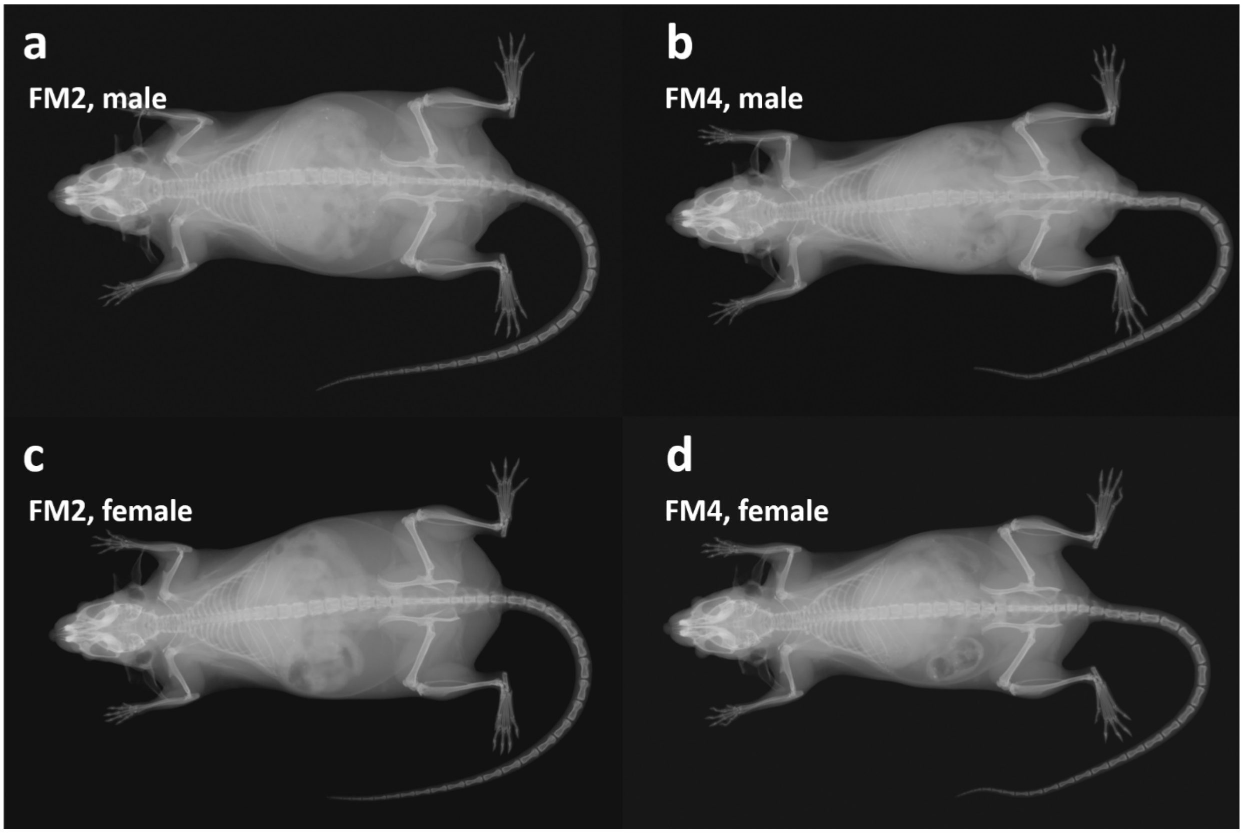

S5 Fig. Dorsoventral radiographic views of the heaviest male and female mice within FM2 or FM4

Dorsoventral radiographic views of (a) the heaviest male mouse colonized with FM2, (b) the heaviest male mouse colonized with FM4, (c) the heaviest female mouse colonized with FM2, and (d) the heaviest female mouse colonized with FM4.

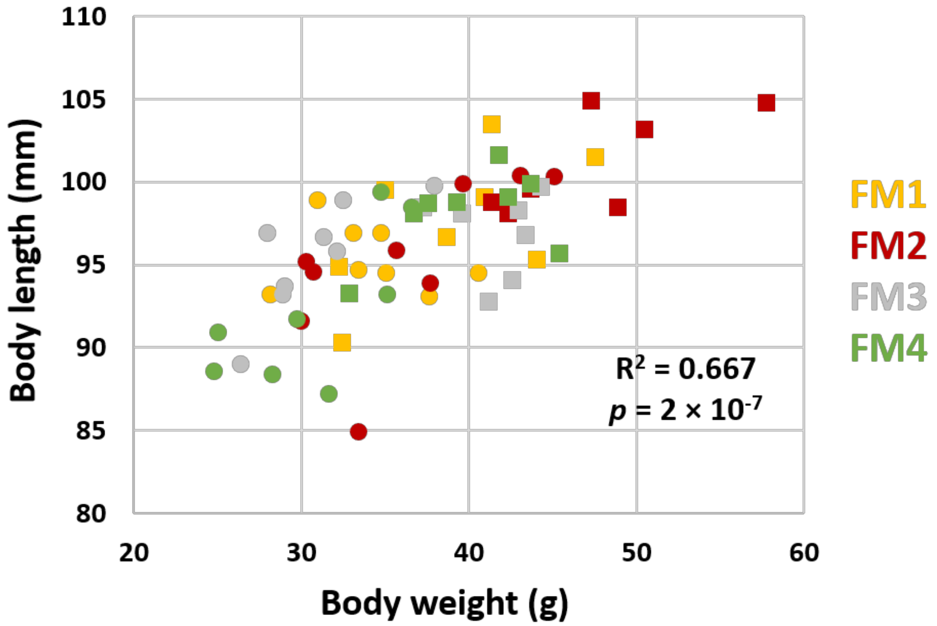

S6 Fig. Correlation between body weight and body length

Dot plot showing the significant correlation between body weight (g) and length (mm) at 16 weeks of age, with circles (female) and squares (males) color-coded by FM (legend at right). Correlation coefficient (R^2) and *p* value based on Spearman's rank order correlation.
