# Supplementary material for: Supplier-origin mouse microbiomes significantly influence locomotor and anxiety-related behavior, body morphology, and metabolism
Source: Commun Biol. 2021 Jun 10;4:716. doi: 10.1038/s42003-021-02249-0 (PMC8192786; doi:10.1038/s42003-021-02249-0)
Supplement: Supplementary file 3 — Description of Supplementary Data [file 42003_2021_2249_MOESM3_ESM.pdf]

Description of Additional Supplementary Files

File name: Supplementary Data 1

Description: This supplemental dataset lists all 16S rRNA amplicon sequence variants (ASVs) found to differ significantly between supplier-origin microbiomes.

File name: Supplementary Data 2

Description: This supplemental dataset shows all data related to the reported behavioral and physiological phenotyping assays.
