# Supplementary material for: Supplier-origin mouse microbiomes significantly influence locomotor and anxiety-related behavior, body morphology, and metabolism
Source: Commun Biol. 2021 Jun 10;4:716. doi: 10.1038/s42003-021-02249-0 (PMC8192786; doi:10.1038/s42003-021-02249-0)
Supplement: Supplementary file 6 — Reporting Summary [file 42003_2021_2249_MOESM6_ESM.pdf]

nature research

Corresponding author(s): Aaron Ericsson

Last updated by author(s): May 17, 2021

Reporting Summary

Nature Research wishes to improve the reproducibility of the work that we publish. This form provides structure for consistency and transparency in reporting. For further information on Nature Research policies, see our Editorial Policies and the Editorial Policy Checklist.

Statistics

For all statistical analyses, confirm that the following items are present in the figure legend, table legend, main text, or Methods section.

n/a	Confirmed	
☐	☒	The exact sample size (*n*) for each experimental group/condition, given as a discrete number and unit of measurement
☒	☐	A statement on whether measurements were taken from distinct samples or whether the same sample was measured repeatedly
☐	☒	The statistical test(s) used AND whether they are one- or two-sided<br>*Only common tests should be described solely by name; describe more complex techniques in the Methods section.*
☐	☒	A description of all covariates tested
☐	☒	A description of any assumptions or corrections, such as tests of normality and adjustment for multiple comparisons
☐	☒	A full description of the statistical parameters including central tendency (e.g. means) or other basic estimates (e.g. regression coefficient) AND variation (e.g. standard deviation) or associated estimates of uncertainty (e.g. confidence intervals)
☐	☒	For null hypothesis testing, the test statistic (e.g. *F*, *t*, *r*) with confidence intervals, effect sizes, degrees of freedom and *P* value noted<br>*Give P values as exact values whenever suitable.*
☒	☐	For Bayesian analysis, information on the choice of priors and Markov chain Monte Carlo settings
☒	☐	For hierarchical and complex designs, identification of the appropriate level for tests and full reporting of outcomes
☒	☐	Estimates of effect sizes (e.g. Cohen's *d*, Pearson's *r*), indicating how they were calculated

Our web collection on statistics for biologists contains articles on many of the points above.

Software and code

Policy information about availability of computer code

Data collection	Some custom R code was used, and is available upon request to readers.
Data analysis	Software used to analyze sequencing data include cutadapt, QIIME2, and DADA2, all of which are cited in the manuscript.

For manuscripts utilizing custom algorithms or software that are central to the research but not yet described in published literature, software must be made available to editors and reviewers. We strongly encourage code deposition in a community repository (e.g. GitHub). See the Nature Research guidelines for submitting code & software for further information.

Data

Policy information about availability of data

All manuscripts must include a data availability statement. This statement should provide the following information, where applicable:

- Accession codes, unique identifiers, or web links for publicly available datasets
- A list of figures that have associated raw data
- A description of any restrictions on data availability

All 16S rRNA sequencing data have been deposited in the National Center for Biotechnology Information (NCBI), Sequence Read Archive (SRA), and are available as BioProject PRJNA683598.

Field-specific reporting

Life sciences study design

All studies must disclose on these points even when the disclosure is negative.

Sample size	Sample sizes were determined based on preliminary pilot studies capable of detecting differences between two of the tested groups using similar samples sizes (n = 8 mice/sex/GM). The fact that we were able to detect so many GM-dependent differences validates our samples size as sufficient.
Data exclusions	No data were excluded from the study.
Replication	While we have not replicated the battery of phenotyping tests reported in the manuscript, we and other collaborators have replicated components of the reported testing, yielding highly concordant microbiota-dependent differences in locomotor and anxiety-related behavior.
Randomization	Mice used for phenotypic testing were bred in one of four colonies a the University of Missouri, and pups were sent to collaborators for phenotyping at 5 weeks of age, until the goal of 8 male and 8 female mice from each colony had been shipped. All pups were shipped as they became available, and no pups were excluded from shipment for any reason, other than having reached the desired sample size for that group.
Blinding	Shipping of mice was handled by colony management staff, per the directions above, and the investigators had no direct role in these processes or the selection of subjects from each colony.

Reporting for specific materials, systems and methods

We require information from authors about some types of materials, experimental systems and methods used in many studies. Here, indicate whether each material, system or method listed is relevant to your study. If you are not sure if a list item applies to your research, read the appropriate section before selecting a response.

Materials & experimental systems

n/a	Involved in the study	
☒	☐	Antibodies
☒	☐	Eukaryotic cell lines
☒	☐	Palaeontology and archaeology
☐	☒	Animals and other organisms
☒	☐	Human research participants
☒	☐	Clinical data
☒	☐	Dual use research of concern

Methods

n/a	Involved in the study	
☒	☐	ChIP-seq
☒	☐	Flow cytometry
☒	☐	MRI-based neuroimaging

Animals and other organisms

Policy information about studies involving animals; ARRIVE guidelines recommended for reporting animal research

Laboratory animals	We have included the desired information in the manuscript, including the species (Mus musculus), strain (Crl:CD-1), sex (male and female), and age (range from 6 to 16 weeks) of animals used.
Wild animals	Study did not involve wild animals.
Field-collected samples	Study did not involve use of any field-collected samples.
Ethics oversight	All studies were performed under Institutional Animal Care and Use Committee (IACUC) approval, at both performance sites.

Note that full information on the approval of the study protocol must also be provided in the manuscript.
